# Magnetic Somnambulism

**Published:** 1867-08

**Authors:** Wm. Mason Turner

**Affiliations:** Philadelphia


### Magnetic Somnambulism.

Translated from the French of Nysten.

BY WM. MASON TURNER, M. D., OF PHILADELPHIA.

Somnambulism is an affection of the cerebral functions characterized by a kind of an aptitude to repeat during sleep those actions which are contracted by habit, either in wandering about or in executing different movements, of which, however, on awakening, there remains no recollection whatever. Somnambulism is, perhaps, a physiologic state or condition, a degree more exalted than the ordinary fantasies of slumber, rather than a nervous affection.

*Magnetic Somnambulism.*—This is a peculiar nervous condition, into which we can throw, by a sort of mental influence, individuals of a high nervous sensibility—particularly hysterical women.—When somnambulism is provoked artificially, the most singular phenomena are observed. Some feel the hallucinations of sight, some of hearing, some of odor, etc., and are falsely made to believe in a transposition of the senses which does not exist. In somnambulism we see sometimes the pathetic faculties, intellectual and moral too, acquire a wondrous development. The memory attains an astonishing precision, and thoughts are delivered in a correct and elegant language.

The theory of this mass of phenomena is clearly cleared up by a knowledge of the physiology of the brain, but loses beyond that all that appears marvellous in it, when we have recourse to the state of scientific facts. We know that in a condition of the most mental harmony, that our internal images are dependent on our external sensations; there is a complete subordination of abstract contemplation to direct observation, and to employ here a trite but very just phrase, *we see things as they are.* But it is demonstrated that even in persons gifted with a superior judgment, it is possible by purely artificial means to develop a cerebral condition in which the *within* takes the place of the *without*, and they are made to behold things otherwise than they really exist. This confirmed mental alienation is nothing but a persistence of that condition, in which we make, in the observed phenomena, the most complicated hypothesis. For a long time it was customary to attribute certain conditions, it may be physiologic or it may be pathologic, to the influence of demons. In the witcheries of magic, as in the science [?] of magnetism, it is necessary to choose well the subject in whom you would produce cries, convulsions, dreams, and ecstacies. Only those practices are otherwise considerably more dangerous than the magnetism, for the former often end by developing *demono-mania.* We can conceive then easily, that a belief in good and evil genii was well calculated to strike with awe, feeble minds.

In the case of somnambulism, a person having been declared proper to exercise the magnetic influence, and for the rest, being inclined by his education to these corresponsive beliefs, familiarizes himself with the administration of the pretended magnetic fluid. Once his technical apprenticeship over, he commences the practice of magnetism, and after a short while, his simple appearance is sufficient to produce profound emotion. In every case, it is easy where one is of strong convictions, and where there are few with whom to deal; for generally it is a matter of no trouble to attract to those who are undecided.

Now this attitude, or that gesture, or these movements, are nothing more than artifice, by means of which there is developed in a person suitably prepared, a cerebral condition more or less decisive, and which can be carried even to that ecstacy which characterizes magnetic sleep. In this condition, moreover, much less frequently to be observed than in simple lethargy, the belief or demi-belief has a power so wonderfully developed in the mind of the patient—of abstract images, of such an intensity, that all direct observation is entirely lost. *General sensibility* can even be annihilated in consequence of this profound interior absorption, and as the meditative organs commence again to exercise themselves on the products of abstract contemplation, the enrapt one can effect a series of ratiocinations sufficiently coherent; and the more, if the auditive impressions continue to operate, there can be established between the magnetiser and the magnetised a connection strongly marked; but in the case of the real ecstacy, the responses of the subject are as vague as those of the Sybil, and in the midst of his devotions the magnetiser interprets them always to the great admiration of his *coterie.*

The convulsive phenomena explain themselves still more easily than do those of somnambulism. When we have studied the procedures of Mesmer, we know how it is that *natural* causes have produced these convulsions. If we wish to consider seriously the veritable cures performed by magnetisers, we will find that they have the same value as the cures of sympathetic medicine, and that cures are performed with the magnetic fluid, as Pyrrhus cured ailments of the spleen by friction made with a *toe of the right foot*, an invention which he shares with Vespasian. The curative power of magnetisers is then a simple illusion, and therein we can here confront two classments of therapeutics which have for each other the greatest affinities. While the magnetiser cures one fluid with another, we have the Homœopaths, who cure the ideal of a disease with the ideal of a remedy. Moreover, nothing should excuse a general system of treatment which enforces, in persons of feeble mind, chimerical beliefs. So the proceedings of magnetisers should be proscribed in therapeutics at once as valueless, and as nuisances. The magnetic fluid administered in *one* day, they say, would be but a very small fraction of an universal fluid, by means of which there is established (according to the theory of magnetisers) a mutual influence between the celestial, terrestrial, and animate bodies.

In going back to the beginning of abstract theories, we find a similar essence, which, under the same name, or that of *love of the world*, serves to bind again our human knowledge, and especially to quench that desire which would explain *all things.* The ease which one has, then, to deceive certain minds, relates not solely to the property which we have, to show without our internal emotions, under any sufficient influence; it rests on the profound scientific ignorance in which the mass of individuals are plunged.

In the phenomenon of the turning tables, we must believe that the table can turn without muscles, without nerves; that it can speak without the organs of voice. But all that is nothing by the side of the rapping-spirits, through the medium of which, every scientific opinion, even the very arches of mathematic phenomena, are shaken. That which contributes again in a great number of cases to the success—happily transient—of these fantastic exhibitions, is that it is not rare to encounter among these believers and propagators, persons instructed in the sciences. But that should only prove one thing, that judgment and common sense, are independent of literary and scientific attainments. Flint, and then Schiff, have indeed shown, in their experiments on the inventors of these juggleries, that the sounds which they produced, were due to a slight displacement (previously occasioned) of the patella—to the tibia on the femur—or to the tendon of the long peroneus, all jerked suddenly into proper position. This displacement is effected by muscular contractions which are easily acquired. Aided by this physiologic knowledge, it has been an easy matter to baffle their trumpery, by causing them to place the limb in a position, in which muscular contraction was impossible. As for this magnetic fluid, there exists nothing, as we see, but an hypothesis denuded of all proof.

Finally, all that interest, which, according to some authors, should appertain to the physiologist, in the study of magnetism, rests in an habitual ignorance concerning the physiology of the brain—and reduces itself to this, that it is easy enough to place such or such an individual, at first, and then an assembly in whole or in part, in an intellectual condition such that the information more or less vague obtained, of the first, are interpreted by the other in the sense which is desired should be contrary to that to which attention has been directed. It is in such a cerebral condition that is to be found, the explanation of all the singular effects of magnetism, the abstractions occasioned by the juggleries which surround us—the changing effects following the practice of magnetism—all dependent on the cerebral condition of the magnetised.

